# Trans-cranial opening of the blood-brain barrier in targeted regions using a stereotaxic brain atlas and focused ultrasound energy

**DOI:** 10.1186/2050-5736-2-13

**Published:** 2014-08-04

**Authors:** Chenchen Bing, Michelle Ladouceur-Wodzak, Clinton R Wanner, John M Shelton, James A Richardson, Rajiv Chopra

**Affiliations:** 1 Department of Radiology, University of Texas Southwestern Medical Center, 5323 Harry Hines Blvd, Dallas, TX 75390-9061, USA; 2 Department of Internal Medicine, University of Texas Southwestern Medical Center, 5323 Harry Hines Blvd, Dallas, TX 75390-9061, USA; 3 Department of Pathology, University of Texas Southwestern Medical Center, 5323 Harry Hines Blvd, Dallas, TX 75390-9061, USA; 4 Department of Molecular Biology, University of Texas Southwestern Medical Center, 5323 Harry Hines Blvd, Dallas, TX 75390-9061, USA

**Keywords:** BBB opening, Focused ultrasound, Stereotaxic system, Drug delivery

## Abstract

**Objective:**

The blood-brain barrier (BBB) protects the brain by preventing the entry of large molecules; this poses a major obstacle for the delivery of drugs to the brain. A novel technique using focused ultrasound (FUS) energy combined with microbubble contrast agents has been widely used for non-invasive trans-cranial BBB opening. Traditionally, FUS research is conducted with magnetic resonance imaging (MRI) guidance, which is expensive and poses physical limitations due to the magnetic field. A system that could allow researchers to test brain therapies without MR intervention could facilitate and accelerate translational research.

**Methods:**

In this study, we present a novel FUS system that uses a custom-built FUS generator mounted on a motorized stereotaxic apparatus with embedded brain atlas to locally open the BBB in rodents. The system was initially characterized using a tissue-mimicking phantom. Rodent studies were also performed to evaluate whether non-invasive, localized BBB opening could be achieved using brain atlas-based targeting. Brains were exposed to pulsed focused ultrasound energy at 1.06 MHz in rats and 3.23 MHz in mice, with the focal pressure estimated to be 0.5–0.6 MPa through the skull. BBB opening was confirmed in gross tissue sections by the presence of Evans blue leakage in the exposed region of the brain and by histological assessment.

**Results:**

The targeting accuracy of the stereotaxic system was better than 0.5 mm in the tissue-mimicking phantom. Reproducible localized BBB opening was verified with Evans blue dye leakage in 32/33 rats and had a targeting accuracy of ±0.3 mm. The use of higher frequency exposures in mice enabled a similar precision of localized BBB opening as was observed with the low frequency in the rat model.

**Conclusions:**

With this dedicated small-animal motorized stereotaxic-FUS system, we achieved accurate targeting of focused ultrasound exposures in the brain for non-invasive opening of the BBB. This system can be used as an alternative to MR-guided FUS and offers researchers the ability to perform efficient studies (30 min per experiment including preparation) at a reduced cost in a conventional laboratory environment.

## Background

For most therapeutic agents aimed at treating central nervous system (CNS) diseases and disorders, the blood-brain barrier (BBB) is a primary physiological barrier limiting drug delivery into the brain parenchyma. The BBB is a separation present along all capillaries in the CNS that controls the molecular transport and diffusion across these blood vessels. The barrier is formed by layers of cells that are coupled by tight junctions [1,2]. Only small-molecule drugs with high lipid solubility and a low molecular mass under 400–500 Da can cross the BBB in pharmacologically significant amounts, hence excluding most current therapeutic and imaged agents from being used in the brain [3,4].

Non-invasive localized opening of the BBB has been demonstrated using focused ultrasound (FUS) exposures combined with circulating intravascular microbubble ultrasound contrast agents. The exact physical mechanisms governing the interactions between the microbubbles and endothelial cells are not known, but it is likely that when stimulated by ultrasound energy, oscillation of microbubbles produces mechanical effects induced by radiation force and/or shear stress on the blood vessel walls, temporarily opening the BBB without tissue damage [5]–[7]. This combination of FUS and intravascular microbubbles offers a unique method for remotely actuating mechanical energy at the site of small vessels throughout the brain. Meanwhile, this opening occurs at lower acoustic power levels than was previously used without intravenous microbubbles, which makes this method substantially easier to apply through the intact skull [8]. As early as 2001, Hynynen et al. demonstrated that focused ultrasound combined with gas bubbles can open the BBB transiently in rabbits [9]. In 2002, Mesiwala et al. confirmed that high-intensity focused ultrasound is capable of a selective and non-destructive disruption of the BBB in rats [10]. Subsequent studies confirmed the feasibility of non-invasive localized BBB opening in rodents as models of human disease [11,1,13]–[16]. However, the small size of rodent brains makes FUS experiments challenging, necessitating the development of dedicated small-animal exposure systems [17].

In the majority of studies to date, confirmation and visualization of BBB opening has been achieved using magnetic resonance imaging (MRI). T1-weighted imaging is often used to confirm successful delivery of gadolinium-based MR contrast agents across the BBB, and T2-weighted imaging can evaluate the presence of tissue damage [18,19]. The major drawbacks of MR-guided FUS are the need for MRI-compatible systems, lack of availability, limited throughput of experiments, and expense. MR-guided focused ultrasound systems are not usually accessible to neuroscientists or other researchers outside of the imaging research field. A method for performing trans-cranial BBB opening without MR guidance would be desirable in order to lower the barrier to entry in this field and to achieve broader penetration of FUS technology in neuroscience.

Previous studies have incorporated systems for BBB opening outside the MRI environment. One method for targeting specific regions in the brain is the use of stereotaxy in combination with an anatomical atlas. This method is widely used in neuroscience to the injection of agents or implantation of electrodes in specific regions of the rodent brain. Liu et al.'s group developed a pinhole-assisted mechanical scanning device using a stereotaxic apparatus for BBB opening [13], and Konofagou et al. applied a stereotaxic frame for localized BBB opening in rodents [20]. Another method for imaging BBB opening is multi-photon fluorescence microscopy. The two-photon microscopy allows *in vivo* visualization of the cerebral vasculature and neurons at the subcellular level [21]. Besides, cerebrovascular dynamics and kinetics of dye leakage after FUS sonication can also be imaging with multi-photon microscopy [22]. However, in order to enable targeting of the ultrasound focus to a specific functional area in the brain, MR images are still required in these systems.

In this study, we present a compact stereotaxic-FUS system to perform trans-cranial localized BBB opening in rodent models using FUS energy and a stereotaxic system with a built-in rodent brain atlas. The goal of this system is to provide a tool for neuroscientists to achieve non-invasive targeted BBB opening in rodents for behavioral and functional research. Using the rodent brain atlas, researchers can easily target a specific functional area of the brain. In this paper, we introduce our stereotaxic-FUS system, followed by initial accuracy characterization in tissue-mimicking phantoms and rodents. Successful BBB opening was confirmed through gross imaging, histopathology, and MR imaging. By all forms of measure, repeatable non-invasive localized BBB opening by our rodent stereotaxic-FUS system was verified.

## Methods and materials

### Focused ultrasound system and stereotaxic apparatus

Ultrasound was transmitted into the brain using a focused transducer with a 25-mm diameter and a 20-mm radius of curvature. The fundamental frequency of the transducer was 1.06 MHz, and the third harmonic frequency was 3.23 MHz, as measured with an impedance analyzer (Via Bravo, AEA Technology Inc., Carlsbad, CA, USA). The dimensions of the ultrasound focus and the pressure output of the transducer were characterized using an acoustic hydrophone tank. A needle hydrophone with a 0.2-mm active area (HGL-0200, Onda Corporation, Sunnyvale, CA, USA) was scanned in three dimensions to measure the spatial pressure distribution of the ultrasound beam at the focus for each transmission frequency.

The transducers were connected to a custom-built compact driving system comprised of an arbitrary waveform generator (LMS271D, Vanuix Corp., Haverhill, MA, USA), an RF amplifier (NP Technologies, Newbury Park, CA, USA), high-power low-pass filters, and a dual-frequency matching circuit that enabled efficient transmission of power to the transducer at each operating frequency. This self-contained driving system was controlled from a laptop computer via USB. One of the main goals was to produce a portable system for focused ultrasound that could be easily transported for experiments.

A standard stereotaxic apparatus (51730 M, Stoelting Co., Wood Dale, IL, USA) was used to achieve targeting of ultrasound energy within the brain with an attachment capable of registering the rat brain atlas (Neurostar, Tubingen, Germany). These systems are normally used to achieve precise insertion of electrodes or injection of materials directly into target regions of the brain. The method of registering the brain atlas to the rodent involves locating the bregma and lambda on the rodent skull sutures with a metal pointer. Once the *x*, *y*, and *z* coordinates of these two locations are identified, the brain atlas (which is referenced to these points) is immediately registered to the animal. Tilting correction was performed automatically based on the current animal to take the difference in brain position and size into consideration.

### Tissue-mimicking phantom

In order to test the spatial accuracy of ultrasound delivery using the stereotaxic-FUS system, a series of exposures were performed in a custom-made tissue-mimicking phantom. The phantom was comprised of a hydrogel with dissolved bovine serum albumin (BSA), similar to previous recipes [23], which could be used to capture the location of the ultrasound focus through coagulation of the BSA. Briefly, gellan gum (1% *w*/*v*, Gelrite, CP Kelco, Atlanta, GA, USA) and salt (0.23% *w*/*v*) were added to and dissolved in deionized, degassed water (90% *v*/*v*). Metamucil (0.18% *w*/*v*, P&G, Cincinnati, OH, USA) was added to the mixture to create ultrasound scattering. BSA (25% *v*/*v*, CF-0020, Boval, Cleburne, TX, USA) was dissolved in the gel to provide ultrasound absorption. BSA also provides an optical marker of the ultrasound focus since the protein undergoes coagulation when heated above 70°C and creates a visible region of opacity within the otherwise transparent gel. The heating of the gel was achieved using a continuous delivery of ultrasound at the high third harmonic frequency (3.23 MHz). The recipe for the tissue-mimicking phantom is shown in Table 1. The speed of sound and ultrasound attenuation coefficient of the phantom material were characterized using a hydrophone tank and were measured to be 1,518 ± 2 m/s and 0.3 ± 0.1 dB/cm (at 1 MHz), respectively.

**Table 1:** Spatial accuracy characterization with the phantom

**Axis**	**Desired (mm)**	**Measured (mm)**	**Error (%)**	**Desired (mm)**	**Measured (mm)**	**Error (%)**
	Target A-B			Target A-C		
*x*	0.5	0.5 ± 0.01	±2	1	1 ± 0.01	±1
*y*	0.5	0.5 ± 0.01	±2	1	1 ± 0.01	±1
*z*	0.5	0.5 ± 0.01	±2	1	0.9 ± 0.01	-2 ~ 0
	Target A-D			Target A-E		
*x*	1.5	1.5 ± 0.01	±0.6	2	2 ± 0.01	±0.5
*y*	1.5	1.5 ± 0.01	±0.6	2	2 ± 0.01	±0.5
*z*	1.5	1.4 ± 0.01	-1.3 ~ -0.6	2	1.9 ± 0.01	-1 ~ -0.5

The spatial accuracy of ultrasound delivery was evaluated by exposing targets with different spacing (0.5, 1, 1.5, and 2 mm) in all three directions (*x*, *y*, *z*). The tissue-mimicking phantom was placed in the position where the rodent head would be located, and the ultrasound transducer was coupled to it using degassed water. After the exposures, the distance between the regions of coagulation in the gel were measured and compared with the desired separation. Tests were completed in two planes (*x*-*y* and *x*-*z*). For each plane, one reference target (A) and four testing targets (B, C, D, E) were selected to apply the sonication.

As an important next step, ultrasound exposures were delivered to the tissue-mimicking phantom in a three-dimensional (3D) pattern based on the rat brain atlas in order to cover a spatial region equivalent to the right motor cortex of the brain. The geometric fidelity of this ultrasound exposure was evaluated in comparison to the brain atlas.

### Animal experiment

#### ***Animal preparation***

**Figure 1:**
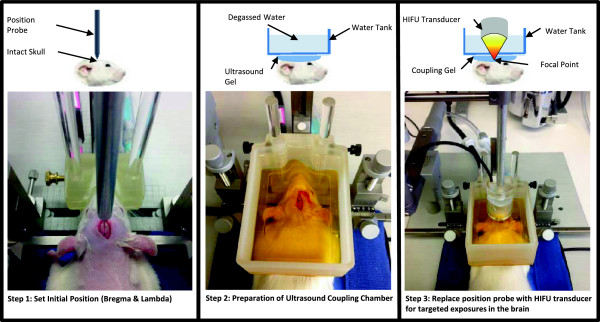
**Overview of the procedure used to perform trans-cranial ultrasound exposures using a standard stereotaxic positioning system.** Step 1: A skin incision is made to expose the skull surface, and a metal pointer is used to locate the bregma and lambda sutures. Step 2: An ultrasound coupling chamber is placed over the skull with a thin layer of ultrasound gel between the chamber and skull. The chamber is filled with degassed water. Step 3: The metal pointer is replaced with a focused ultrasound transducer whose focus is now aligned with bregma. The rat brain atlas is used to determine the appropriate distance to move the ultrasound focus to a targeted region in the brain.

Female 200–270-g Sprague Dawley rats (*n* = 56) and female 20–25-g Swiss Webster mice (*n* = 14) were used in this study. All procedures were approved by UT Southwestern Institutional Animal Care and Use Committee and followed guidelines set forth by the Guide. Animals were anesthetized with a mixture of 2%–3.5% isoflurane and 1–2 l/min of 100% oxygen. A 24G I.V. catheter was placed in the lateral tail vein. A pulse oximeter was attached to the animal's paw to monitor heart rate and oxygen saturation, and a rectal temperature probe (attached to a homeothermic control blanket) was used to record and maintain core body temperature (PhysioSuite, Kent Scientific Corp., Torrington, CT, USA). Hair over the cranial surface of the skull was removed using an animal trimmer and depilatory cream (VEET sensitive formula, Reckitt Benckiser, Parsippany, NJ, USA). After preparation, the animal was transferred to the stereotaxic apparatus and stabilized using ear bars and bite bar. A custom-built nose cone was placed over the animal's nose to deliver inhalant anesthetic.In order to perform brain atlas registration, a skin incision over the skull was performed to identify cranial landmarks bregma and lambda. Ultrasound gel was applied on the skull, and a custom-built water reservoir filled with degassed water was lowered over the skull for ultrasound gel coupling. The ultrasound probe was mounted on the stereotaxic system followed by target selection from the brain atlas. The ultrasound probe was then automatically moved to the targeted area to generate a focus at the specific location. This setup procedure is described in Figure 1.

Evans blue (2%; 3 ml/kg rats, 4 ml/kg mice) was injected via the tail vein catheter and allowed to circulate for a minimum of 1–3 min. A bolus injection of microbubbles (30 μl/kg rats, 60 μl/kg mice, Optison, GE Healthcare, Milwaukee, WI, USA) was delivered via the tail vein catheter, with simultaneous start of ultrasound sonication. When multiple sonications were performed (in the same animal), 5 min of wait time was applied to allow microbubbles to clear from circulation.

All brain targets were selected in the right hemisphere (center of caudoputamen) while the left hemisphere was kept as a control. Twenty-three rats were utilized to optimize FUS parameters (frequency, microbubble dosage, focal pressure, etc.) on BBB opening results. After optimization, 33 rats were utilized to verify the reliability of the system and to characterize spatial accuracy. Among those rats, 5 were utilized for histology. Nineteen mice were utilized to optimize ultrasound frequencies specific to mouse cranial space and parietal bone thickness (7 for low frequency, 1.06 MHz, and 12 for high frequency, 3.23 MHz).

Animals were sacrificed and perfused 5–10 min after sonication with sterile saline and 10% buffered formalin, followed by immediate harvesting of the brain. Brain samples were then placed in 10% buffered formalin to allow for 24–48 h of fixation time. Brains were sliced using a rat or mouse brain matrix (World Precision Instrument, Sarasota, FL, USA) to confirm localization of Evans blue leakage following BBB opening. Photographic record of Evans blue leakage was made, and a subset of subgross slices was worked up by histology.

#### ***Histological preparation***

Subgross coronal brain-matrix slices from perfusion-fixed animals were paraffin processed, embedded, and sectioned according to standard procedures [24,25]. Serial paraffin sections were prepared at the epicenter of FUS-mediated hemorrhage, seen by dark-field microscopy [26]. Resulting sections were stained by hematoxylin and eosin (H&E) and terminal deoxynucleotidyltransferase-mediated UTP end labeling (TUNEL). H&E-stained sections were analyzed for histopathologic hallmarks of brain injury (hemorrhage, edema, nuclear condensation, inflammatory cell infiltrate, rarefaction). Histologic analysis for possible ultrasound damage to neuronal and glial cell populations in the FUS target area was performed via TUNEL; nuclei of apoptotic and necrotic cells were labeled with fluorescein according to methods of the first report [27]. Sections subjected to TUNEL were counterstained with propidium iodide.

#### ***Ultrasound exposures***

Single-frequency pulsed exposures (10-ms burst duration) were transmitted into the brain with a repetition frequency of 1 Hz and a total exposure duration of 120 s. In rats, the intracranial peak negative pressure at the focal point under low frequency (1.06 MHz) was adjusted to be between 0.5 and 0.6 MPa, based on the hydrophone calibration of the transducer and published measurements of the insertion loss through rat parietal bone [28]. Similar opening is expected with lower pressure. The threshold for the BBB opening was estimated to be inversely proportional to the square root of frequency [29]. Prior studies have investigated extensively the effects of different sonication parameters on the threshold of BBB opening [18]: frequency [30], pulse width [31,32], pulse repetition time [31], total sonication duration [33], microbubble size [34], and contrast agent dose [35,36]. The ultrasound exposure parameters selected for this study have been shown to achieve consistent opening of the BBB in rodent models [37]. For the mouse exposures, a 3.23-MHz frequency was used instead of 1.06 MHz in order to achieve a relatively localized ablation within the mouse brain. Skull insertion loss under high frequency is still unknown, so it was assumed to be approximately 50% based on the skull thickness. The focused ultrasound energy was adjusted to achieve a focal pressure of 0.5–0.6 MPa accordingly.

#### ***Characterization of in vivo targeting accuracy***

Evans blue dye has been used frequently as an indicator of BBB opening. Evans blue binds to albumin which cannot cross the BBB; therefore, neural tissue remains unstained after intravenous administration of the dye [38]. In these experiments, BBB opening was confirmed on gross tissue sections based on the leakage of Evans blue dye in the targeted area of the brain.

As a secondary validation, two rats that received ultrasound exposures to open the BBB in a targeted region were administered an intravenous injection of gadodiamide (0.02 mmol/kg, Omniscan, GE Healthcare, Milwaukee, WI, USA) and were imaged in a 9.4 T animal imaging and spectroscopy system (Avance, Varian Medical Systems, Palo Alto, CA, USA). Contrast-enhanced T1-weighted spin-echo MR images were obtained to visualize BBB opening through diffusion of the contrast agent into the brain parenchyma (field of view 128 × 128, TR/TE 151/10 ms, slice thickness 1 mm, gap 0 mm, echo number 1).

The spatial targeting accuracy of the system was evaluated through localization of the region of Evans blue staining on gross sections. Two specific targets were manually selected using the brain atlas, and the spacing between targets was measured on the gross sections. Atlas spacing was compared with measured spacing in three axes: anterior-posterior (AP), medial-lateral (ML), and superior-inferior (SI). Reference in ML and SI axes is the midline of the brain and top surface of the brain, respectively. For characterization of the AP axis, a reference point was selected as coordinates 0.00, 3.32, and 5.55 mm (AP, ML, and SI) relative to bregma since the cranial landmarks disappear after removal of the skull. Five rats were utilized to characterize targeting accuracy along each axis.

## Results

### Focused ultrasound transducer characterization

**Figure 2:**
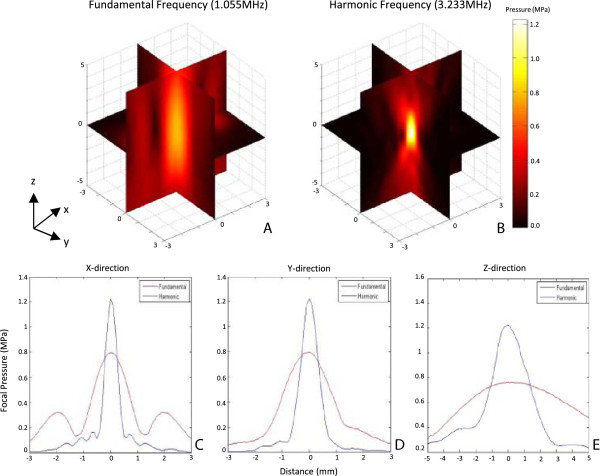
**The ultrasound beam plots. (A, B)** 3D beam plots for the fundamental and the third harmonic frequency, respectively. At the third harmonic frequency, the focal area is more compact with higher intensity. **(C-E)** Intensity profiles in three directions (*x*, *y*, *z*) for the two frequencies. The peak amplitude could reach 0.25 V at the fundamental frequency (*red curve*) while 0.38 V at the third harmonic frequency (*blue curve*).

The spatial pressure distribution produced by the focused ultrasound transducer, driven at the fundamental and the third harmonic frequency, was characterized in an acoustic hydrophone tank. The pressure distributions are shown in Figure 2 as a 3D map of the ultrasound beam. The scan was completed in planes of 6 × 10 mm^2^ along (*x*-*z*, *y*-*z*) and 6 × 6 mm^2^ transverse (*x*-*y*) to the beam to cover the entire focal area. Two sidelobes along the *x* direction were caused by electrode tabs on the transducer. The ultrasound transducer was excited with a 20-cycle pulse (40 cycles at the third harmonic frequency) and 100-Hz repetition frequency signal. The pressure at each location was acquired as an average of 256 pulses. The full width at half maximum (FWHM) of the beam was measured to be 1.6 × 2 × 10 ± 0.1 mm (*x* × *y* × *z*) for the fundamental frequency and 0.4 × 0.6 × 3 ± 0.1 mm (*x* × *y* × *z*) for the third harmonic frequency. The beam volume is approximately 0.0336 and 0.00072 cm^3^, respectively. At higher frequency, the focal area is about 46 times more compact. The pressure profile along each axis (*x*, *y*, *z*) is shown in Figure 2. The peak negative pressure at the focal point ranged from 0.1 to 0.8 MPa at the fundamental frequency and from 0.05 to 1.2 MPa at the third harmonic frequency.

### Spatial accuracy characterization—phantom

**Figure 3:**
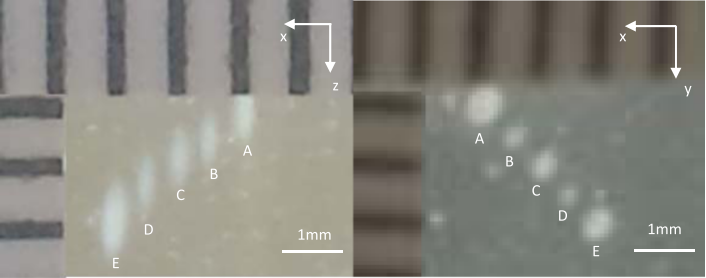
**Isolated regions of coagulated BSA within the phantom caused by focal heating with the 3.23-MHz transducer.** The test was performed in the *x*‒*z* direction (*left*) and the *x*‒*y* direction (*right*) to measure the separation between individual sonications. The precision of targeting was approximately 0.01 mm, and the accuracy was less than 2%. The greatest error occurred in the *z* direction which is due to the difference in speed of sound between the coupling water and phantom and the different amounts of each in the beam path with different focal depths.

Figure 3 shows a sample pattern of coagulated volumes generated in the tissue-mimicking phantom. The desired separation and measured separation for each target are included in Table 2. In the *x* and *y* axes, the precision of targeting was approximately 0.01 mm for four different separations. However, in the *z* axis, the measured spacing was smaller than the desired spacing by up to 2%. This was likely due to the difference in ultrasound attenuation and sound velocities between the phantom and water.

**Table 2:** Spatial accuracy characterization with the rat model

	**Atlas spacing (mm)**	**Measured spacing (mm)**					**Percentage error**
		**Rat A**	**Rat B**	**Rat C**	**Rat D**	**Rat E**	
Medial/lateral	3.32	3.5	3.1	3.0	3.1	3.1	±6.02
	4.03	4.2	4.4	4.0	4.1	3.9	±4.96
Superior/inferior	5.55	5.1	5.4	5.7	5.7	5.7	±3.78
	4.37	4	4.5	4.1	4.5	4.3	±4.44
Anterior/posterior	3	3.2	3.1	2.9	2.9	3.2	±4.67
	6	6.1	5.9	5.8	5.8	6.1	±2.33

**Figure 4:**
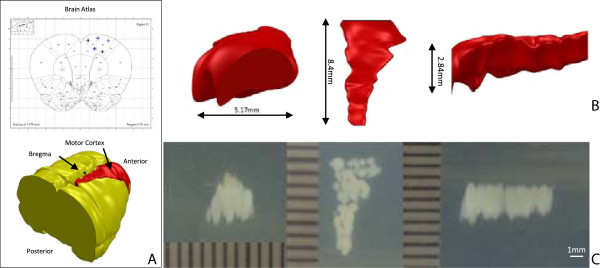
**Results obtained for generating a 3D ultrasound exposure pattern. (A)** A 3D rendering of the right motor cortex (*red*) and the brain (*yellow*), segmented from the standard rat brain atlas. **(B)** Views of the motor cortex from each of the three principal axes. **(C)** The corresponding pattern of coagulated BSA in the gel phantom along the same views and spatial scale. The 3D pattern of coagulation in the gel phantom was equivalent to the shape of the motor cortex, and the volume was within 10%.

Figure 4 shows the results obtained for generating a 3D ultrasound exposure pattern that overlapped with the rat brain right motor cortex. The dimension of the motor cortex model was measured to be 8.4 × 2.84 × 5.17 mm (*L* × *H* × *W*), with 123.34-mm^3^ volume. The dimension of the phantom coagulation was measured to be 8 × 3.4 × 4.6 ± 0.1 mm (*L* × *H* × *W*), with 125.12 (117.315 ~ 133.245)-mm^3^ volume. The percentage error of pattern volume was within ±10% (-4.88% ~ 8.03%).

### Animal studies

#### ***Validation***

**Figure 5:**
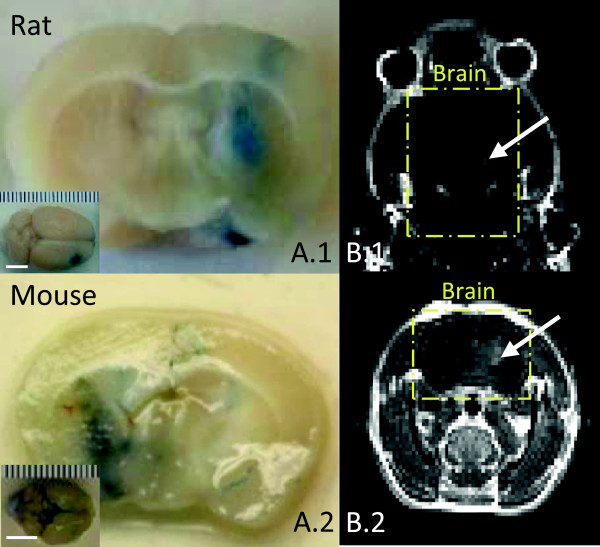
**Validation with the animal model.** Evans blue dye leakage served as primary validation. *A.1*/*A.2* shows BBB opening on the rat/mouse separately. A photo of the entire brain is referred at the corner; *bar* is 5 mm. T1‒weighted spin‒echo multi‒slice MR images (TR/TE, 151/10 ms) were acquired with rat as secondary validation. *B.1* and *B.2* show images in transverse and coronal directions, respectively. *Yellow rectangle* highlighted the brain, and contrast enhancement (*white arrow*) indicates localized BBB opening.

Evidence of successful and confined BBB opening following FUS was observed by both Evans blue dye leakage and contrast-enhanced MR images in the targeted brain area. Figure 5(A.1) shows the whole brain and targeted brain slice for rat, under low-frequency (1.06 MHz) sonication. Evans blue dye can be observed on the cortical brain surface and in the corresponding underlying slice, indicating the location of BBB opening. On the other hand, when low frequency was also applied in mice for comparison, Evans blue dye leakage was observed through the entire targeted hemisphere, likely due to the thinner parietal bone. In order to achieve a similar tight focus, high frequency (3.23 MHz) was applied in the mouse model. Figure 5(A.2) shows the BBB opening results for mouse. In both the rat and mouse models, the un-targeted hemisphere presents as control. Figure 5(B) renders the T1-weighted MR images acquired as secondary validation. B.1 refers to a transverse slice while B.2 refers to a coronal slice. The presence of an increased signal in the brain due to extravasation of the contrast agent indicates localized BBB opening.

#### ***Spatial accuracy characterization — animal***

**Figure 6:**
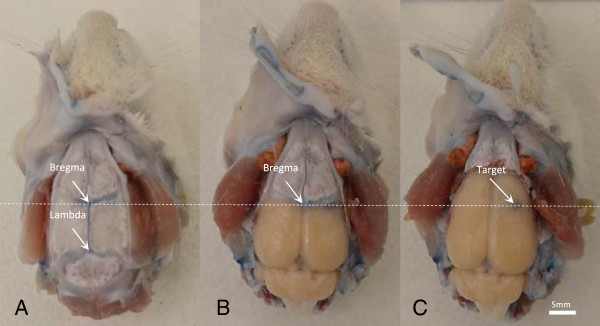
**Overview of the dissection procedure applied to verify the accuracy of the reference target.** The location of the reference target is (0, 3.32, 5.55), measured based on bregma. Brain suture and landmarks are shown in **(A)**, whole brain with the skull removed is shown in **(C)**, and **(B)** serves as an in‒between photo. *White dash line* performs as an indicator of bregma.

The system's spatial accuracy relative to the brain atlas was characterized in three axes: anterior/posterior (AP), medial/lateral (ML), and superior/inferior (SI). Figure 6 shows a stepwise overview of brain registry as the calvarium was removed. Photos were taken before and after removing the calvarium to indicate the location of bregma and the reference point. Validation results in three axes are included in Table 2. Atlas spacing was acquired from the brain atlas during the targeting procedure. Measured spacing was acquired from the brain slice after dissection, relative to references. The location of each exposure was manually selected to be at the centroid of the focal area. Percentage error was calculated based on average measured spacing and atlas spacing. Percentage error for targeting is estimated to be around ±5%.

#### ***Histology***

**Figure 7:**
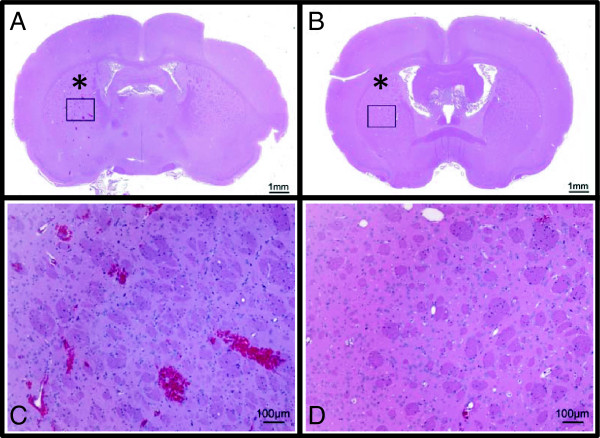
**H&E-stained sections for acute (A, C) and 72‒h recovery (B, D) rats.** Both rats received the same ultrasound exposures in the same location in the brain, indicated by an *asterisk*. Low-magnification images of the entire brain section (A, B) depict the exposed and control hemispheres. A cutting artifact is observed in (A) on the right cortical region and is unrelated to the ultrasound exposure. A × 10 magnified region (inset in A and B) depicts the cellular architecture in the exposed regions of the brain. Evidence of perivascular hemorrhage in the target region can be observed in the acute exposure.

**Figure 8:**
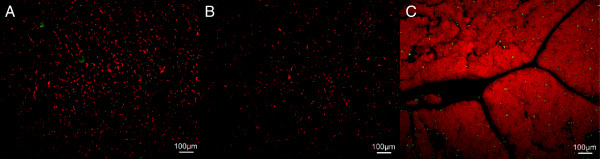
**Tissue sections stained with propidium iodide ( *red *) and TUNEL ( *green *) for acute (A) and 72-h recovery (B) rats.** The sections were taken from the same regions of the brain as are shown in Figure 7C,D. The absence of green staining in the acute and recovery rats demonstrates that no cells with nicked DNA were present in these regions. An image of juvenile mouse thymus TUNEL-positive control **(C)** is included at the same magnification for contrast to the slight green autofluorescence of extravascular red blood cells present in the brain sections exposed to ultrasound.

As further validation of the rat model, detailed histologic analysis was performed on coronal-matrix slices collected immediately following FUS and 72 h post-FUS exposure. Figure 7 shows ultrasound-induced BBB opening by routine histopathology. H&Es of acute and 72-h brains both show evidence of perivascular hemorrhage in the target region. Hemorrhage was limited to the target area in both lateral-medial and anterior-posterior axes. Mild perivascular edema and condensation of adjacent neuronal nuclei was sparsely evident. Low-magnification images illustrate the contralateral hemisphere without hemorrhage.In the absence of major histopathology on H&E in FUS rat brains, TUNEL was performed to look for apoptotic and/or necrotic vascular endothelium and the surrounding brain parenchyma. No cells with nicked DNA were detected in the brains from either acute or 72-h post-FUS rats (Figure 8). Images of juvenile mouse thymus TUNEL-positive control are included in the figure for contrast to the autofluorescence of extravascular RBCs present in FUS brain sections.

## Discussion

This study presented a novel system that interfaces a stereotaxic brain atlas and a FUS energy transducer to achieve non-invasive localized BBB opening in rodents, without MR intervention. The spatial accuracy of the system was estimated to be ±2% with the tissue-mimicking phantom and ±5% with the rodent models. Evans blue dye leakage was used as a subgross indicator of localized BBB opening, which was subsequently validated by intact animal MR imaging of gadolinium contrast agent. Applying low frequency (1.06 MHz) and high frequency (3.23 MHz) on rats and mice, respectively, a tight three-dimensional focus of ultrasound was achieved. This portable system can be used for rodents' BBB opening in a traditional laboratory environment.

The primary research application we envision for this system is for the targeting of regions within the rodent brain to facilitate functional neuroscience studies in rodents. Since the majority of these investigations are conducted in conventional laboratory environments in close proximity to behavioral testing facilities, a portable FUS system that does not require MRI targeting and guidance is desirable. Furthermore, the brain atlas is an important targeting tool for functional regions of the brain in rodents.

To our knowledge, this is the first study to explore the use of high-frequency FUS exposures in mice for BBB opening. Exposure of the mouse brain at 1.06 MHz resulted in a very large region of BBB opening in the brain encompassing the entire AP direction within a hemisphere. This is due to the thinner parietal bone and smaller brain size in mice. Exposures at 3.23 MHz achieved a localized region of BBB opening similar in proportion to the brain as was achieved using 1.06 MHz in rats. However, unlike experiments with the rat model, the skull insertion loss is still unknown at this high frequency, and the focal pressure inside the mouse brain was estimated for this study based on prior measurements at a lower frequency in the rat skull [28]. Experimental measurement of the insertion loss through the mouse skull bone at 3 MHz is necessary to obtain more accurate estimates of the intracranial pressure amplitudes during these exposures.

The system described in this manuscript still suffers from a few limitations. In order to achieve BBB opening within a larger region of the brain, rapid mechanical [17] or electronic [39] steering of the ultrasound focus is desirable. The stereotaxic system described in this study is amenable to these technical improvements, and this addition would enable regional opening of target structures within the brain. The incorporation of acoustic feedback has been demonstrated to achieve more consistent stable cavitation during ultrasound exposures for BBB opening [40,41]. Due to the lack of imaging confirmation of BBB opening with the stereotaxic system, this type of feedback would be an important improvement to the FUS system. Another limitation of this approach is that an incision is needed to identify bregma and lambda. Potential strategies to overcome this would be to use the external ear bars as a reference point or to use a technique like high-frequency ultrasound imaging to non-invasively locate the appropriate stereotactic reference point on the skull. These approaches will be the subject of future investigations. Nonetheless, while these improvements would expand the capabilities of the existing system, the current configuration is still capable of consistent opening of the BBB in rodents.

## Conclusion

A novel system that interfaces a stereotaxic brain atlas and a FUS transducer for non-invasive targeted BBB opening in rodents is presented in this study. The spatial targeting accuracy of the system was estimated to be ±2% in tissue-mimicking phantoms and ±5% in the rat brain. Localized opening of the BBB was achieved at 1.06 MHz in rats and 3.23 MHz in mice. Consistent opening of the BBB was verified through Evans blue staining in gross brain sections and histopathology in H&E-stained tissue sections. TUNEL staining confirmed that there was minimal to no apoptosis nor necrosis in the exposed regions of the brain.

## Competing interests

The authors declare that they have no competing interests.

## Authors' contributions

CB was the main generator of this manuscript. She was responsible for developing and characterizing the stereotaxic focused ultrasound system. She also worked as an operator during the animal experiments for this study. MW was the veterinary technologist and preclinical coordinator in this study. She led the animal experiments involved in this project. She also contributed to the writing of the animal study section in this manuscript. CRW, JMS, and JAR performed and analyzed the histological tissue sections produced in this study. They also co-wrote the histology section in this manuscript. RC was the principal investigator of this study. He came up with the scientific concept and overall design for the study and reviewed the manuscript. He also supported the scientific work financially from existing grant support. All authors read and approved the final manuscript.
